# Editorial to the Special Issue: “Dysregulation of Human Molecular and Metabolic Mechanisms Resulting in Oxidative Stress and Damage Generation in the Space Environment”

**DOI:** 10.3390/ijms23126466

**Published:** 2022-06-09

**Authors:** Thomas J. Goodwin, Melpo Christofidou-Solomidou

**Affiliations:** 1 Goodwin BioScience Research Institute, Houston, TX 77058, USA; 2 Sovaris Aerospace, Research Innovation, Infectious Disease Research Center Colorado State University, Fort Collins, CO 80521, USA; 3 The National Aeronautics and Space Administration (NASA Retired) Johnson Space Center, Houston, TX 77058, USA; 4 Pulmonary, Allergy, and Critical Care Medicine, Department of Medicine, University of Pennsylvania Perelman School of Medicine, Philadelphia, PA 19104, USA; melpo@pennmedicine.upenn.edu

## 1. Introduction

Commercial space industries are emergent, bolstered by new exciting rocket systems, orbital and landing vehicles, the creation of multi-country orbital platforms, satellite technology, the renewed promise of low Earth orbit (LEO) business opportunities, as well as promised planetary exploration [1]. The anticipation of Moon and Mars landings by remote vehicles and advertised human exploration overwhelm the space news of the day [2]. Yet the excitement we fear is reaching a crescendo, which foreshadows outcomes fated to result in profound disappointment if specific, critically important human factors and systems’ physiology are not methodically addressed. While the advent of mechanical and digital machine technology has leapt ahead, disproportionately little attention has been devoted to the human protoplasm that will reside within these magnificently engineered vehicles [3]. Here, we will review the particular salient points of and challenges to human health maintenance in extraterrestrial environments and how those factors will ultimately govern the success of humankind to enact plans to work in and colonize space.

What are these health “challenges” or “factors” obstructing the critical pathway to our realization of space business industrialization and new world colonies? Answering this question requires the assessment of a fundamental imbalance in the human physiology observed in space flight and reduced gravity environments. The “imbalance” is one of the known common denominators in physiological regulation, which is oxidative stress and damage (OSaD) elicited by reactive oxygen and nitrogen species (RONS) [4,5,6]. Indeed, contributions to this syndrome may stem from concomitant hyper-exposure to several toxic factors, including the explicitly increased high energy radiation, reduced gravity or microgravity (μG), hypoxia and hyperoxia conditions observed in each and every extravehicular activity (EVA), and the inevitable inhalation of Moon and Mars dust and/or regolith attendant to prolonged planetary exploration [4,5,6,7]. These first two factors (radiation and μG) continue to be routinely investigated and considerable data have been accumulated in the popular and scientific literature, reflecting the impact on the OSaD coefficient via genomic, proteomic, and metabolomic responses [3,4,5,6,7,8,9,10,11]. However, variances in gas content and pressures, and the induction of potentially toxic dust/regolith particles into the physiology has to date not been interrogated significantly in the research literature, especially in the context of the combinatorial threat of all these major factors impacting the physiology simultaneously. To address multiple OSaD factors in chorus with one another, the choice of research model is critical and certainly the likelihood that multiple models may need to be employed is warranted. The current scientific literature documents have extensively employed rodent and human physiological platforms to evaluate OSaD generated toxic effects, spanning many different physiological and analytical subsystem models [6,7,8,9,10,11,12,13,14,15,16,17]. 

In the remainder of this editorial, we will discuss the critically important intersection and space health implications of three of the four aforementioned factors and the rationale for concern, specifically increased radiation hypoxia and hyperoxia and dust/regolith ingestion. 

We will first examine the effects of changing oxygen concentrations and pressures within the context of EVAs and planetary explorations. As we know, EVAs are essential to a survey mission profile, and thus unavoidable for the Moon and Mars. NASA’s plans for design reference missions (DRMs), the framework of how astronauts will actually accomplish planetary investigations, continues to evolve predicated on space suit and manned rover design, the methodologies for incorporating meaningful exploration with realistic work cycles, habitat design and function, and the documented limits of human performance and endurance [4]. The current DRMs forecast planetary living in habitats at ~7.6 psi with a pO_2_ of 125 mmHg or in a slightly hypoxic environment (normal pO_2_ is 159 mmHg). However, when conducting EVAs, the suit environment will be ~4.3 psi with an enriched pO_2_ of 222 mmHg, which is hyperoxic [4,7] and each of these conditions are known to lead to the generation of RONS, as discussed in Schmidt and Goodwin’s work in 2013 [4]. The rationale for the aforementioned pressures and O_2_ concentrations stems from the following two requirements: (1) to speed the acclimation and reduce “pre-breathe” time before EVA; and (2) low pressures in suit operations are required to maintain limb and digit dexterity for manual work [4,17]. The current DRMs dictate cyclic changes in these conditions at 48–72 h intervals for multiple months on end. Thus, the operational protocols as they presently exist precipitate a unique source of RONS, which we predict will increase in severity, based on the frequency of programed exploration events on any planetary surface. 

One of the most understudied but potentially catastrophic toxic RONS generators is the virtually assured assimilation of Moon and Mars dust/regolith during prolonged exploration missions. As an Earth-based correlate, if we examine the indices and accounts of coal particle inhalation, we find significant cause for concern. Black lung disease, “miner’s lung”, has long been associated with early death in below ground coal miners, but a more appropriate parallel is the health consequences of airborne particulate matter (PM) in environmental proximity to mountain top mining (MTM) [18,19]. We believe that understanding how coal PM permeates the human physiology sheds light on potential dust/regolith hazards. Although course and ultrafine coal particles are clearly a danger to lung function and a long-term cancer risk, their most important menace may well be the ability to translocate into the central nervous system (CNS) by way of two less obvious conduits, specifically crossing the blood–brain barrier via the ophthalmic and trigeminal neural pathways located in and along the nasal cavity and of course the bronchio-tracheal airway [19,20,21]. Indeed, the data indicate that ultrafine (Nano) particles of coal dust PM_2.5_ to PM_10_ (2.5–10 μM) do access the CNS and result in damaging effects associated with increases in reported cases of dementia, Alzheimer’s and Parkinson’s diseases [18,19]. Concordant with these particle infiltrations is the documented cellular hypersensitivity and neuro-inflammation associated with the aforementioned degenerative diseases of the CNS [20,21,22,23,24]. The metabolomic and biochemical catalysts for RONS generation in the human physiology includes the following infiltrated molecules found in ultrafine coal dust: SiO_2_ (silica or silicates), the primary driver for the syndrome of silicosis or black lung plus varying concentrations of an array of other reactive metals, including numerous species of Si, Fe, Ti, Mg, Mn, Zn, Ni, V, Cr, Cu, Pb, Cd, Sb, Se, As and Sn, which depending on the concentration are known to be severe health risks [25,26]. Certain metallic elements from the larger group (Si, Cu, Sb, Sn, Pb, Zn, As and Ni) can be aerosolized during MTM operations into airborne PM2.5 fractions, which are deeply inhaled, leading to the most bioreactive fraction size. These elements are expected to be mainly present in the form of arsenides, antimonides silicates, and oxides [19,25,26].

Interestingly, when we inspect the content of Moon and Mars dust/regolith, we find a disturbing number of resemblances, risks, and possible hazards not unlike fine coal dust. An analysis by Lemmon et.al. in 2004, performed in conjunction with the Mars Exploration Rovers missions, determined that fine particulate dust on Mars is curiously similar to fine coal dust particles. Mars fine dust/regolith particles are ~1–4 μM and have an atmospheric particles average of ~1–2 µM in diameter [27]. As previously stated, fine coal particles’ PM are in the range of 2.5–10 μM so size is ominously comparable [18]. One added factor that theoretically adds a heightened hazard risk is the significantly reduced gravity of the Moon and Mars environments. Both Moon and Mars exhibit lower gravitational acceleration coefficients, so the size of PM that remains suspended will tend to be smaller and thus “concentrate” undesirable particle fractions that are likely to be hazardous [17,27,28]. Quoting directly from Krisanova et al. in 2013, the “major elemental composition of Lunar Dust (LD) simulant (JSC-1a, Lunar Soil Simulant) (in %): SiO_2_ (46.67), TiO_2_ (1.71), Al_2_O_3_ (15.79), Fe_2_O_3_ (12.5), FeO (8.17), MnO (0.19), MgO (9.39), CaO (9.9), Na_2_O (2.83), K_2_O (0.78), P_2_O_5_ (0.71). Composition of Mars Dust MD simulant (JSC Martian Soil Simulant) (in %): SiO_2_ (34.5), TiO_2_ (3), Al_2_O_3_ (18.5), Fe_2_O_3_ (19), FeO (2.5), MnO (0.2), MgO (2.5), CaO (5), Na_2_O (2), K_2_O (0.5), P_2_O_5_ (0.7)” [28]. A quick comparison revealing the close elemental match between LD and MD simulants is perhaps not too surprising. In addition to the elemental species listed above for LD and MD, LD also contains nickel (Ni), chromium (Cr), and MD contains toxic levels of chlorine (Cl), chromium (Cr), and sulphur trioxide (SO3) [26,27,28]. However, the comparison between the coal dust elements above, known to result in severe disease, and LD and MD is enlightening and alarming. 

Attempting to forecast the potential hazards to astronauts from LD and MD and the comparison to ultrafine coal PM2.5 show that several toxic components are present in ultrafine coal dust, LD and MD, which are known health hazards, i.e., Si, Fe, Ti, Mg, Mn, Zn, Cr, Cu, Ni and are substantial constituents in all three types of dust. Of special note, the concentrations of SiO_2_, which is the major contributor of silicosis in miner’s black lung, are LD 46.67%, MD 34.5% and dependent on the type of coal being mined between 2–10% [22,23,24,25,26,27,28]. Even to the casual observer, a red flag from these data should be the distressingly similar concentrations of SiO_2_, a known silicosis hazard and the resulting diseases that occur from coal dust on Earth. Furthermore, the concentrations of SiO_2_ in LD and MD are remarkably higher; therefore, we suggest on this basis alone that there should be greater concern for RONS in these exploration environments regarding dust/regolith. 

It is unfortunate that each of the four aforementioned physical challenges resulting in considerable RONS generation and metabolic turmoil do not exist as “one offs”. In that arena, they might be more easily addressed and overcome. This, however, is not the case, rather they are inextricably tied to one another based on individual genomics, metabolism and biochemistry [6,7,17]. As we postulate the associated impact of the entire interwoven space habitation environment, we are confronted by at least one imperative pivotal threat, RONS/OSaD, and the question of how we can mitigate systemic imbalance. Enumerating the sources, we can conclude that reduced gravities, changing atmospheric gas and pressure concentrations, increased galactic cosmic radiation (GCR) and LD and MD dust/regolith are serious threats. To demarcate more completely the RONS threat, we need to consider the interaction of reduced gravity, LD/MD and GCR in particular. Although seemingly unrelated, the likely occurrence of heavy metals in the blood stream and neural tissues of the astronauts could be catastrophic [28,29], especially in long duration missions. 

**Figure 1:**
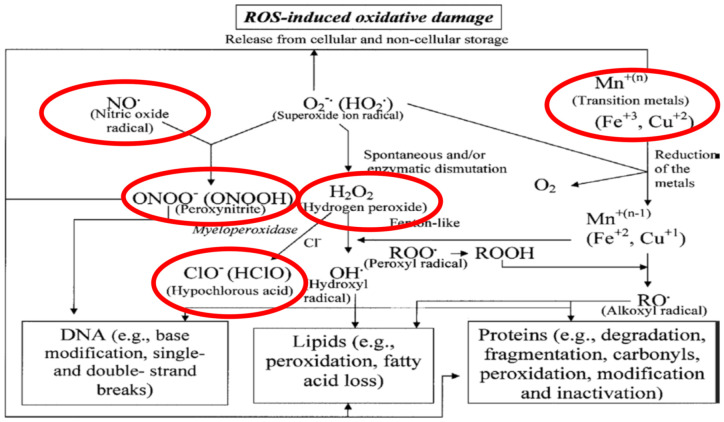
Reproduced with permission from [6] published by Sage Journals, 2002.

Kohen and Nyska in 2002, as shown in Figure 1, defined several species of ferric, manganese, and copper molecules, which serve as resident catalysts for RONS production, such as nitric oxide, hydrogen peroxide, peroxynitrite, hypochlorous acid, and perchlorate molecules, which damage DNA, lipids, and proteins [6], especially when potentiated by high energy radiations (HZE), such as the case with GCR.

**Figure 2:**
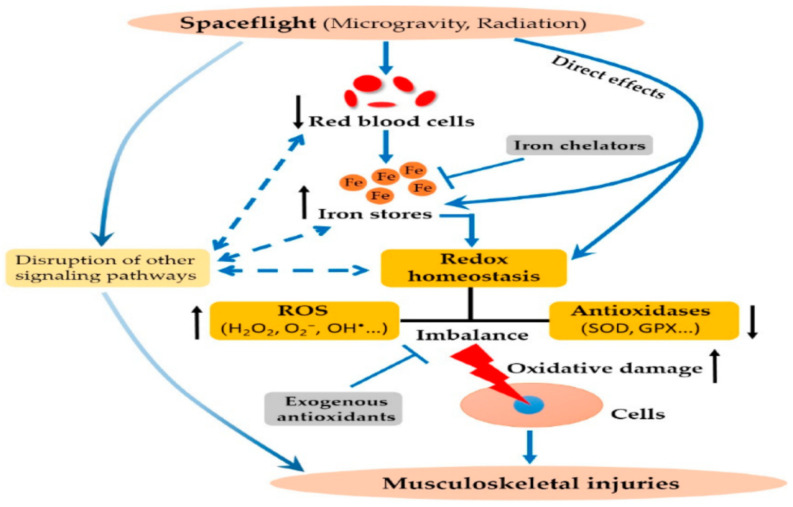
Credit to [29].

Yang et. al. in 2018, as shown in Figure 2, measured serum ferritin (SF) in astronauts and verified iron overload increases found by Zwart et al. in 2013 [29]. Increased SF is known to precipitate bone and muscle loss [29] without the presence of LD or MD. As detailed by Krisanova et al. in 2013, LD and MD PM is assisted in traversing the alveolar lung surface through the interaction of Al_2_O_3_, attaches to the nerve terminals (synaptosomes) in the CNS, and results in increased glutamate binding, manifested via the presence of FeO, Fe_2_O_3_, and MnO, leading to the disruption of neural homeostasis and improper brain biochemistry in the physiology. This single challenge alone portends serious malfunction, even in the absence of the other three challenges enumerated above [28].

Our final challenge to discuss is the effect of GCR on mammalian and human CNS cells. A great deal of excellent work has been accomplished in the last 12 years, illuminating the heretofore unrealized fragility resident in the mammalian brain. Klein et al. in 2021 and Krishnan et al. in 2021 described how even low dose neutron radiation through the generation of RONS damages the normal operation of the CNS and that chronic low dose exposure negatively effects the hippocampus and the prefrontal cortex, due to altered neurotransmission [14,15]. These studies illuminate the disruption of cellular processes at the DNA and molecular genetic levels [15,16,17]. The central flaw in the previous work of the space radiation community at large was the amount and manner of administration of radiation in acute protocols used in the attempts to mimic actual GCR. The career permissible exposure limit (PEL) found in the NASA Technical Standard 3001 accepts that astronauts will receive as much as 0.3 Gy during a yearlong tour on the International Space Station (ISS) and up to 1.5 Gy to the CNS during a lifetime career dose [30,31]; thus, some previous experimental studies employed from 0.1 to 5.0 Gray (Gy) irradiations. Recently, however, the next step in our understanding of the vulnerability of brain/CNS function has been accomplished by demonstrating that continuous and remarkably low doses of radiation, i.e., 0.18–0.30 Gy, results in an appreciable loss of cognitive function in the mammalian brains of rats and mice [32,33]. If these new detrimental threshold values are reflective of the same “damage” in humans, then an extraordinary problem exists as the prevailing expectation is that astronauts will receive a cumulative GCR radiation dose of at least 0.6 to 1.15 Gy during a 2.5 to 3.0 year mission to Mars [30,31]. These newly realized data are far below the PEL and previously conceptualized expectations.

In summary, the four toxic challenges/factors reviewed above—specifically increased high energy radiation (GCR), reduced gravity or microgravity, hypoxia and hyperoxia conditions—observed in each and every EVA and the inevitable inhalation of extremely small Moon or Mars dust and/or regolith PM linked to prolonged planetary exploration [4,5,6,7,8,18,19,20,21,22,23,24,25,26,27,28] constitute a potentially lethal stew in the context of extended exploration missions. Noteworthy research by a plethora of world-class scientists has been conducted in good faith over the last 35 years in an attempt to disentangle and mitigate these challenges, and yet the challenges remain largely unresolved. Clearly, the classically employed reductionist approach to dealing with these complexities has realized only marginal success over substantial years, not to the disparagement of the research participants. The inherent difficulties in modelling these complex conditions simultaneously are incontrovertible, yet our ineradicable position is effective countermeasures will only follow all-inclusive investigational strategies. Therefore, scientists worldwide should be encouraged to embrace a physiological universalism at the molecular and “omic” levels. This includes an envisioned approach to addressing chronic space RONS imbalance and the resultant OSaD as a realistic pivotal target for advancing space habitation.
